# Contribution of Academic Satisfaction Judgments to Subjective Well-Being

**DOI:** 10.3389/fpsyg.2022.772346

**Published:** 2022-05-20

**Authors:** Mauricio F. Zalazar-Jaime, Luciana S. Moretti, Leonardo A. Medrano

**Affiliations:** ^1^Secretaría de Investigación, Universidad Siglo 21, Córdoba, Argentina; ^2^Departamento de Psicología, Pontificia Universidad Católica Madre y Maestra, Santiago de los Caballeros, Dominican Republic

**Keywords:** subjective well-being, academic satisfaction, satisfaction judgments, university students, structural equation modeling

## Abstract

The role of academic satisfaction (AS) on persistence and successful academic behavior has been the focus of research for decades. Nevertheless, driven by positive educational psychology, subjective well-being has been highlighted as another central feature in the academic path of students. Studies aimed at identifying the variables that contribute to explain different aspects of academic performance have been widely investigated, although studies aimed at identifying the determinants of subjective well-being are still limited. The present paper examined the contribution of AS judgments on subjective well-being (SWB). To this end, it was hypothesized that SWB levels depend on the balance between positive/negative emotions and life satisfaction judgments. Furthermore, it was stipulated that AS has an indirect contribution on SWB, through life satisfaction, whereas the balance of emotions influences both AS judgments and life satisfaction. Using an analysis strategy based on structural equation modeling, the results indicated that the model fitted satisfactorily, explaining 32% of the variance of SWB. Particularly, it was observed that AS judgments contributed to life satisfaction judgments (β = 0.34). Although no direct contribution of AS on SWB was reported, a total contribution partially mediated by life satisfaction judgments was revealed (total β = 0.19). These findings support the importance of academic satisfaction judgments, not only because of their importance in academic terms, but also because of their impact on university students’ subjective well-being and health.

## Introduction

### Contribution of Academic Satisfaction Judgments to Subjective Well-Being

Positive education promotes the development” of educational environments that allow students to learn an established educational curriculum, while acquiring the skills necessary to develop their own well-being and that of others (Oades et al., 2011; Riedel et al., 2020). This is a fundamental key point because the development of positive teaching practices and methods can contribute to achieving learning potential, developing positive attitudes toward higher education and skills for future employment of university students (Nilson, 2010; Eryılmaz, 2015).

The pursuit of happiness and achieving “a good life” are considered to be one among the elemental missions of psychology (Povedano-Diaz et al., 2020). However, the scientific study of well-being does not aim to prescribe what a good life entails, but to analyze the factors that lead people to positively evaluate their lives according to their own parameters. This evaluation is called “Subjective well-being” (SWB) and has been shown to be a phenomenon of importance for physical and mental health. Different research indicates that SWB is beneficial for health (Brummett et al., 2009; Segerstrom and Sephton, 2010) and longevity (Lyubomirsky et al., 2005; Howell et al., 2007; Chida and Steptoe, 2008). In addition, SWB is associated with supportive social relationships, citizenship, work performance, and resilience (Lyubomirsky et al., 2005; Diener and Chan, 2011).

SWB is a desirable goal for the society as a whole and in particular for adolescent and young adults (D’Agostino et al., 2019). During this distinctive stage, biological, psychological, cognitive and social changes can affect the levels of SWB, so this variable can be considered as an indicator of how they cope with these changes (Povedano-Diaz et al., 2020). SWB also works as a protective factor, people with higher SWB are more likely to develop health behaviors such as exercising, not smoking and drinking less alcohol (Diener et al., 2018). Also, higher levels of SWB are associated with the ability to form positive relationships, establish strong self-esteem, effectively express feelings and regulate emotions, persevere, and positively engage in challenging tasks (Shoshani and Slone, 2017; Bradshaw, 2019). Different studies also indicate that SWB is related to positive academic outcomes, and a better adaptation to the demands and characteristics of the university system (Casas, 2011; Fernandes Ferreira Lima and Araujo de Morais, 2018; Tomás et al., 2020).

### Subjective Well-Being and Academic Satisfaction in University Students

The most popular model on SWB is the one proposed by Diener et al. (2018), which “includes people’s appraisals and evaluations of their own lives” (p. 1). This conceptualization differentiates, on the one hand, cognitive judgments such as those linked to life satisfaction and on the other hand, an emotional component which involves a balance between positive affect (pleasant and desirable feelings and moods) and negative affect (unpleasant and undesirable feelings and moods). In this model, the role of satisfaction judgments is key. According to Diener et al. (2018) when people reflect on their life, they make both general (considering the totality of their life) and specific judgments (they evaluate more specific domains such as satisfaction with studies, work or family). Goal satisfaction theory (Diener et al., 2018) assumes that the satisfaction of key needs, desires and goals will produce to high levels of SWB, and thus the dissatisfaction of them will produce to low levels of SWB. Various experimental and non-experimental studies indicate that vital satisfaction assessments are strongly associated with SWB indices (Schimmack, 2008) and changes in satisfaction assessments generate significant changes in SWB (Gamble and Gärling, 2012).

As proposed by Diener (1984, 1994) the general assessment of life satisfaction depends successively on the extent of satisfaction within a group of specific domains. In addition, at different points in life, priorities and life circumstances change, and thus we will expect that the predictors of happiness can also change (Jebb et al., 2020). In this sense, it is expected that satisfaction with the academic experience constitutes a domain of great importance for people during their university studies (Lent, 2004).

Academic satisfaction (AS) is a high priority satisfaction domain in the lives of college students (Lent, 2004; Lent et al., 2017). Several studies sustain the importance of AS on well-being and life satisfaction (Garriott et al., 2015; Sheu et al., 2016). A review by Suldo et al. (2006) refers to a series of studies suggesting that students’ feelings and attitudes toward their university are significantly related to their level of life satisfaction. Likewise, studies developed within the framework of social cognitive career theory highlight the contribution of AS on overall life satisfaction, along with other relevant constructs such as self-efficacy beliefs and perception of social support (Lent et al., 2009, 2012; Ojeda et al., 2011; Sheu et al., 2017).

On the other hand, it is important to note that academic dissatisfaction can also be a source of subjective discomfort. During their formative years, university students are exposed to numerous academic demands, which can lead to stress, loss of confidence and demotivation. AS constitutes a protective factor against stress and helps students to cope more healthily with the challenges of academic life (Tessema et al., 2012; Lent et al., 2013; Kong and Yan, 2014). In contrast, it has been observed that students with low levels of AS present greater vulnerability to stress and lower academic performance, which negatively affects their well-being (Kuo et al., 2014).

It should also be noted that the conceptual delimitation of AS has not been without controversy. AS is often understood as a subjective and global cognitive assessment by students of their learning experience at university. AS judgments therefore derive from the comparison students make between their expectations and their perceived academic experience, the conformation of those assessments being supported by the knowledge available within the autobiographical memory (Bickart and Schwarz, 2001). As pointed out by Lent et al. (2007), there is a parallel conceptualization that understands AS as “the level of enjoyment that students perceive when carrying out experiences linked to their role as students” (p. 87). However, this definition is considered inadequate since positive affect would be a variable closely linked to, but different from satisfaction judgments. Positive affect influences the shaping of satisfaction judgments (Schoefer, 2008) and would also increase as a consequence of the favorable evaluation that people make (Tessema et al., 2012). This close link between positive affect and satisfaction judgments leads to the fact that they are often used interchangeably, when in fact they are associated but different variables. In the present study, AS is considered as a cognitive construct linked to, but distinct from, affect.

**FIGURE 1:**
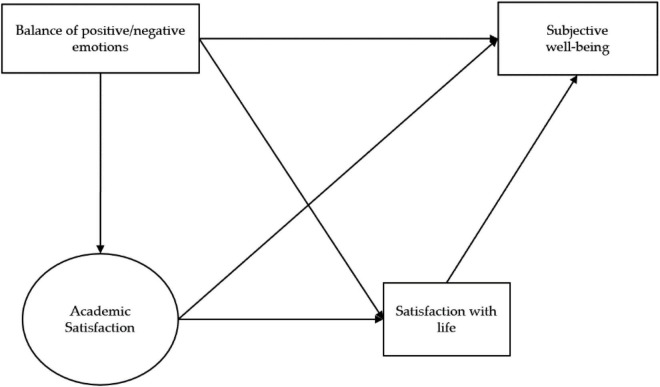
Contribution of academic satisfaction on the subjective well-being model.

Despite the relevance of AS on student well-being, most of the studies conducted have focused on investigating the role of AS on academic behavior (e.g., academic performance and persistence). In fact, no SWB studies specifically addressing the contribution of AS to SWB were found within the literature. For this reason, the objective of this study was to evaluate the impact of academic satisfaction on the SWB of university students. Specifically, and according to the model proposed by Diener, we hypothesized that: (a) levels of subjective well-being depend on life satisfaction judgments and the balance of positive/negative emotions; (b) domain-specific judgments (academic satisfaction) have a direct and indirect contribution, through life satisfaction, on SWB; and (c) the balance of emotions influences academic satisfaction and life satisfaction judgments (see Figure 1).

## Methodology

### Participants

The sample was constituted by 326 university students between 17 and 46 years old (*M* = 21.76; *SD* = 1.61), of both sexes (men = 38.7%; women = 61.3%). The participants were from public (68%) and private (32%) universities, distributed in the following careers: social sciences (48%), natural sciences (29%), technology (21%) and arts and humanities (2%). The sampling was non-probabilistic and accidental. That is, the possibility of participation was limited to those students who were on the university campus and gave their consent. In addition, as this was a cross-sectional study, no dropouts were recorded.

### Instruments

#### Subjective Well-Being

To measure this variable, Subjective Happiness Scale (SHS; Lyubomirsky and Lepper, 1999) was used. In order to achieve a measure of overall “Subjective Well-being,” Lyubomirsky and Lepper (1999) have designed the SHS to measure the levels of Well-being in a comprehensive and global sense. This measure is a subjective assessment of whether a person is happy or unhappy. This is a 4-item scale, two of the items ask participants to characterize themselves in terms of statements such as “*in general I consider myself*….” on a 7-point Likert scale, where 1 is *not very happy* and 7 is *very happy*. The other two items provide a brief description of happy and unhappy individuals and ask participants to say the extent to which they identify with each description, for example “*some people are very happy in general and enjoy life come what may, exploiting life to the full. To what extent does this characterization describe you*? where 1 is *not at all* and 7 is *to a large extent*. Vera-Villarroel et al. (2011) reported studies on psychometric properties with adequate internal factor structure and consistency (α values between 0.73 and 0.87) for populations of adults, adolescents and university students; *r*-values obtained for depression and neuroticism scales in studies of discriminant validity were between –0.24 and –0.63 and significant positive correlations with measures of optimism and extroversion between 0.27 and 0.55. It should be noted that in this context the concepts of subjective happiness and well-being are equivalent (Lyubomirsky and Lepper, 1999).

#### Positive and Negative Affect Scale

This is a 20-item scale in which 10 items evaluate states of positive affect (for example “active,” “strong,” “inspired”) and the remaining 10, states of negative affect (such as “guilty,” “scared,” “hostile”; Watson et al., 1988). Participants were asked to indicate on a 5-point Likert scale the extent to which they experience each one of the mentioned affective states, 1 signifying *very seldom or never* and 5 *very frequently or almost always*. In his original version, Watson et al. (1988) reported studies with an internal consistency of between 0.86 and 0.90 for Positive Affect and between 0.84 and 0.87 for Negative Affect. In a study adapted to Argentina, Medrano et al. (2015) were able to replicate the structure of two factors and indicated acceptable indices of internal consistency (α = 0.83 for Negative Affect and α = 0.82 for Positive Affect).

#### Satisfaction With Life Scale

This 5-item scale investigates the level of satisfaction with situations related to life in general Diener et al. (1985); participants respond on a 7-point Likert scale where 1 is *completely disagree* and 7 is *entirely agree*. Diener et al. (1985) reported satisfactory internal structure, test-retest reliability and internal consistency (Cronbach’s α = 0.87) for the scale’s psychometric properties. For the present study the version translated into Spanish by Gómez et al. (2007) was used, in which there are 5 response options, with 1 representing total disagreement and 5 total agreement. For the psychometric properties the Spanish version showed adequate values for internal consistency (Cronbach’s α = 0.84), internal structure (exploratory factor analysis) and convergent-discriminant validity (Atienza et al., 2000).

#### Academic Satisfaction

Since the basic aim of the current paper was to evaluate the contribution of AS to the model of SWB, it was decided to treat the former as a latent variable in order to arrive at a more concise measurement. Structural equation modeling (SEM) allows for the simultaneous use of several variables for a theoretical, unobservable construct, which ultimately leads to more valid conclusions on the construct level, helping to reduce measurement error. In the present study we applied three different AS scales. The first, AS1, is the scale developed by Lent et al. (2007) comprising 7 items in which respondents must evaluate their level of satisfaction with different aspects of their academic experience (for instance, “*I enjoy my classes most of the time*”). Respondents are asked to indicate the extent to which they agree to each statement on a 10-point Likert scale. The original psychometric studies suggested that the scale has a unidimensional factor structure and high internal consistency (Cronbach’s α = 0.94). The version adapted by Medrano et al. (2014) was used, in which the internal structure and internal consistency structures are satisfactory (α = 0.85). The second tool (AS2) is the scale developed by Fernandes Sisto et al. (2008) comprising 11 items (statements such as “*I like the classes*”) to be rated on a 4-point Likert scale where 1 is *never* and 4 *always*. For the purpose of the present study, the version adapted by Medrano and Pérez (2010) was used. The psychometric results corroborated the unidimensional structure of the scale *via* exploratory factor analysis and its internal consistency (Cronbach’s α = 0.84). The third instrument, AS3, is the global index of academic satisfaction (Argyle, 1987). This scale consists of a single item assessing academic satisfaction *via* a graphic representation of responses on a visual scale of 10 positions. Students were asked to respond on a scale of 1–10 to the item “*based on how you feel about your studies, indicate where you would place yourself on the following diagram?*” where 1 is “*the worst learning experience I could ever have”* and 10 *is “the best learning experience I could ever have.*”

### Procedure

After providing clear and explicit information to the university authorities concerning the nature and purpose of the study, questionnaires were delivered collectively during class under the supervision of the authors of the paper. The voluntary nature of participation was emphasized and the confidentiality of the data assured, stating clearly that the results would be used exclusively for research purposes and that the identity of the individual participants would remain confidential. Each participating student signed a consent form. The following questionnaires were then handed out over the next 12 min: Subjective Happiness (Lyubomirsky and Lepper, 1999), Positive and Negative Affect Scale (PANAS; Watson et al., 1988), Satisfaction With Life Scale (SWLS; Diener et al., 1985) and the three measures of Academic Satisfaction (Argyle, 1987; Lent et al., 2007; Fernandes Sisto et al., 2008).

SEM was used to examine the relationships among variables. The main advantage of the SEM technique is that it allows for a more precise analysis of empirical data by taking into account latent variables and complex patterns of relationships between variables.

## Results

### Initial Descriptive and Exploratory Analysis of the Data

Atypical univariate cases were identified by calculating the Z ratings for each variable (*Z* > ± 3.29 were considered atypical) and multivariate cases by means of the Mahalanobis distance (*p* < 0.001; Tabachnick and Fidell, 2013). Twenty two atypical univariate cases were observed and 19 multivariate. Although atypical cases tend to distort the results it was decided to include them in the analysis since otherwise the data would fail to represent a segment of the population (Hair et al., 1999). The mean, standard deviation, asymmetry and kurtosis were calculated. Values between ± 1.00 were considered excellent for evaluating the asymmetry and kurtosis indices and values lower than ± 2.00 were considered adequate (George and Mallery, 2016). All asymmetry and kurtosis variables presented values between ± 1.00 with the exception of General Satisfaction, which showed an adequate level of asymmetry R (-1.11), higher than the kurtosis range (2.40; see Table 1). Multivariate normality was verified by Mardia’s coefficient (Mardia, 1970, 1974), giving a value of 12.06, which is below the critical value of 70 suggested by Rodríguez Ayán and Ruiz (2008) and thus approaches the standard normal distribution. The association between the different variables was verified using Pearson’s *r* correlation coefficient. All correlations were statistically significant with weak to moderate *r*-values, thus discarding any overlap between the variables (Tabachnick and Fidell, 2013; see Table 2).

**TABLE 1:** Descriptive statistics for mean and standard deviation, asymmetry and kurtosis of the variables comprising the subjective well-being model.

	Mean deviation	Standard deviation	Asymmetry	Kurtosis
Balance of positive/negative emotions	9.09	9.76	–0.31	–0.18
Satisfaction with life	18.67	3.39	–0.36	–0.39
Subjective well-being	20.64	3.65	–0.45	0.00
Academic satisfaction 1	52.01	9.23	–0.96	1.55
Academic satisfaction 2	54.81	11.86	–0.74	0.92
Academic satisfaction 3	7.68	1.39	–1.11	2.40

**TABLE 2:** Bivariate correlations between the variables comprising the subjective well-being model.

	1	2	3	4	5	6
1. Balance of positive/negative emotions	1	0.34**	0.33**	0.24**	0.17**	0.34**
2. Satisfaction with life		1	0.54**	0.38**	0.26**	0.39**
3. Subjective well-being			1	0.24**	0.17**	0.29**
4. Academic satisfaction 1				1	0.77**	0.62**
5. Academic satisfaction 2					1	0.56**
6. Academic satisfaction 3						1

**p < 0.01.

### Evaluation of the Subjective Well-Being Model

The statistical software Mplus 6.12. was used to assess goodness of fit using the maximum likelihood estimator (ML), applying the following statistical tools to measure the fit of the model: Chi-squared, Comparative Fit Index (CFI), the Tucker-Lewis Index (TLI), Root Mean Square Error of Approximation (RMSEA), and the Standardized Root Mean Square Residual (SRMR). CFI and TLI indices with values between ≥ 0.90 and 0.95 or higher are considered an acceptable to excellent fit and values of < 0.08 are expected for RMSEA and < 0.06 for SRMR (Hu and Bentler, 1999; Yu and Muthén, 2002). The results indicated a satisfactory fit (χ^2^ = 9.176; df = 5; *p* = 0.000; CFI = 0.99; TLI = 0.98; RMSEA = 0.05, [90% CI = 0.00, 0.10], SRMR = 0.020), explaining 32% of the subjective well-being variance.

**FIGURE 2:**
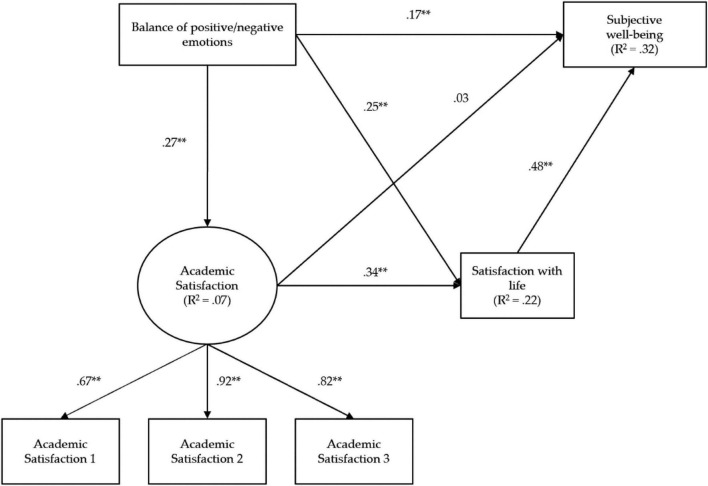
Contribution of academic satisfaction on the subjective well-being model. Standardized path coefficients. **p < 0.01.

Direct contributions of satisfaction with life (β = 0.48, *p* < 0.01) and positive/negative emotions (β = 0.17, *p* < 0.01) were observed. On the contrary, the relationship between academic satisfaction and subjective well-being was not corroborated (β = 0.03, *p* > 0.05). In order to adequately understand how one variable relates to another, indirect effects, understood as the product of the two standardized direct effects involved, must also be considered. By examining these effects it is verified that satisfaction with life modulates the relationship between positive/negative emotions and subjective well-being (indirect effect, β = 0.17, *p* < 0.01). Similar behavior was evident with respect to the contribution of academic satisfaction and subjective well-being through satisfaction with life (indirect effect, β = 0.16, *p* < 0.01). When examining the magnitude of the total effects (see Table 3), it can be seen that the variables that have the greatest contribution to subjective well-being are positive and negative emotions (β total = 0.34), and academic satisfaction (β total = 0.19, see Figure 2).

**TABLE 3:** Total effects, direct and indirect, of the subjective well-being model.

Model variables	Effect		
	Direct	Indirect	Total
**Balance of positive/negative emotions**			
On academic satisfaction	0.27**	–	0.27**
On satisfaction with life	0.25**	0.09**	0.34**
On subjective well-being	0.17**	0.17**	0.34**
**Academic satisfaction**			
On satisfaction with life	0.34**	–	0.34**
On subjective well-being	0.03**	0.16**	0.19**

**p < 0.01.

## Discussion

Many universities are focused on the students’ performance, specifically in terms of good grades, without paying enough attention to other relevant aspects that should be part of the overall personal development (Tomás et al., 2020). According to Diener (1994), levels of well-being depend partially on the degree of satisfaction with different life domains (Argyle, 1992, 1994; Diener et al., 1999; Diener, 2000). The importance of each domain varies in accordance with its proximity and immediacy in a person’s life. AS judgments could thus be an important determinant of the level of well-being of university students; however, current scientific research has been centered on factors associated with academic performance and the specific contribution of AS has not been analyzed (Smith et al., 2003). Academic performance is of course a relevant construct, but high performing students with low SWB present lower levels of well-being (Lent et al., 2009; Tessema et al., 2012; Balkis, 2013; Kuo et al., 2014). This lack of studies taking into account the effect of academic satisfaction on mental health therefore led us to evaluate its contribution in the subjective well-being model proposed by Diener (1994) as applied to a sample of university students in Cordoba, Argentina.

Our findings corroborate the importance of AS judgments in well-being: it was observed that the greater the academic satisfaction, the more likely this was to favorably impact on students’ satisfaction with life in general. No direct and significant influence of satisfaction assessments on well-being was observed, possibly due to the effect of moderators such as age or the introductory/strictly theoretical nature of some subjects, which could bias satisfaction levels. Future studies should therefore control for these variables. Nonetheless it was observed that the total effect was slightly higher than the direct contribution of positive and negative emotions on well-being. These results are consistent with those reported in the literature. In a recent study, Lee and Shin (2022) evaluated a cross-cultural model of academic and life satisfaction, highlighting the contribution of positive affect on AS and the contribution of these constructs on life satisfaction.

In the context of efforts to promote the health and optimal academic behavior of students, Durkin and Joseph (2009) point out the crucial importance of looking more closely into the factors that promote well-being. Intervention strategies based on the theoretical framework of Lent’s Social Cognitive Career Theory (Lent, 2004) would contribute toward explaining how students develop these satisfaction judgments. For example, Lent (2004) reports that in self-efficacy beliefs about one’s ability to perform well, having positive outcome expectations and feeling supported, tend to affect academic satisfaction judgments. Measures to endorse self-efficacy beliefs should therefore be centered on incrementing the capacities involved: establishing and advancing toward a set of performance goals for each stage of academic study, predicting favorable results and perceiving the environment to be supportive.

As mentioned by Diener (1994, 2000) and Lent (2004) and in congruence with the triadic model (taking into account personal, behavioral and environmental factors) proposed by Bandura (1987), the findings of the present study underscore the fact that AS judgments not only affect the academic domain but also have an impact on satisfaction with life and indirectly, on well-being. These results highlight the importance of providing experiences that promote changes in AS. In the literature related to SCCT, it is observed that the most important promoter of satisfaction judgments is self-efficacy beliefs. Different studies (e.g., Wang et al., 2004; Tschannen-Moran and McMaster, 2009) point out the importance of focusing on the sources of information (mastery experience, social persuasion, vicarious learning, and physiological/emotional states), which contribute to such beliefs (Bandura, 1987). In this sense, Lam and Santos (2018) developed an intervention program aimed at modifying self-efficacy beliefs through the identification of appropriate models, anxiety management, recording of negative/destructive thoughts, small group discussions, consultations with the tutor and individual assignments (workbooks), among other aspects. This line of action, added to the recommendations made by Brown and Krane (2000) to modify such sources could be used as a focus to increase beliefs and, therefore, satisfaction judgments.

Regarding positive and negative emotions, the present study observed that students with positive emotions tend to be more satisfied with their lives, inducing a higher level of well-being. The literature linked to positive psychology highlights different intervention practices aimed at increasing positive affect (Sin and Lyubomirsky, 2009). For example, daily recording of gratitude (Emmons and McCullough, 2003; Froh et al., 2008) and recounting one’s own acts of kindness (Otake et al., 2006) has been associated with higher levels of positive affect, life satisfaction, and a decrease in negative affect. Similarly, Seligman et al. (2005) have shown that writing down three good things that went well each day and using the top strengths in a novel way each day for a week, increases well-being and decreases depressive symptoms. As mentioned by Fredrickson (2001) generating practices linked to positive emotions contributes to optimal functioning by increasing the thought-action repertoire, psychological resilience, and emotional well-being, thus minimizing persistent negative emotions.

Another modality of intervention lies in mindfulness practice. Burke (2014) argues that negative affect can be reduced as reactivity is reduced, as a consequence of adopting a non-judgmental perspective on internal experiences; whereas positive affect can increase as a consequence of engaging in the full experience of life, relating to oneself and third parties in a self-compassionate manner. On this point, there are intervention models based on the Mindfulness-Based Stress Reduction (MBSR) paradigm adapted to educational contexts (see Broderick and Metz, 2009; Santorelli and Kabat-Zinn, 2009; Kaiser-Greenland, 2010; Semple and Lee, 2011, for examples), which have evidenced satisfactory results. As indicated by Diener et al. (2018) the different practices aimed at modifying SWB are characterized by persistent effects during moderate times and the effects of their effectiveness may vary depending on the practice performed by individuals. Based on these results, the development of intervention programs should be incorporated into the educational curriculum to be sustainable over time.

As for the limitations of the study, it should be noted that the sample is somewhat small, with a higher proportion of women, and the distribution of students in the different areas of knowledge is not homogeneous, with a greater representation of social sciences. These aspects should be considered for the generalization of the results. However, the review of the literature and, particularly, the theoretical foundations that support the relationships between the variables, suggest the suitability of the model and similar results can be found if the indicated limitations are overcome.

Despite the previous points, suggestions for future lines of research stand out. First, the construct of positive and negative affect could be complemented with an evaluation of the intensity and frequency of these emotions (Diener et al., 1985) to analyze the differential role of these variants. Second, as mentioned in several studies (Argyle, 1992, 1994; Diener et al., 1999; Diener, 2000), the particular circumstances of each student’s life, e.g., family, friends and social relationships, should be assessed to increase the explanatory power of the well-being model. Thirdly, in recent years, the presence of integrative models has been observed in the educational literature, which allow the combination between them, allowing the evaluation of multiple dimensions of educational behavior (Lent et al., 2013). In this sense, a line of action could include the constructs of positive emotions, engagement, relationships, meaning and accomplishment (PERMA) proposed by Seligman (2011) to assess well-being, to the proposed model with the aim of analyzing the interplay between the variables under study. As mentioned by Lee and Shin (2022) and, fourthly, empirical studies of a longitudinal nature are still limited compared to cross-sectional studies. This situation does not allow us to inspect both the temporal prevalence of the constructs and the possible bidirectional relationships. Therefore, longitudinal studies are needed to analyze these aspects. Finally, as a result of the development of these lines of action, it is necessary to carry out intervention programs through the development of practices and/or teaching methods, with the aim of contributing to positive education.

## Data Availability Statement

The raw data supporting the conclusions of this article will be made available by the authors, without undue reservation.

## Ethics Statement

The studies involving human participants were reviewed and approved by Comité Académico de ética en Investigación de la Facultad de Psicología (Universidad Nacional de Córdoba). The patients/participants provided their written informed consent to participate in this study.

## Author Contributions

All authors listed have made a substantial, direct, and intellectual contribution to the work, and approved it for publication.

## Conflict of Interest

The authors declare that the research was conducted in the absence of any commercial or financial relationships that could be construed as a potential conflict of interest.

## Publisher’s Note

All claims expressed in this article are solely those of the authors and do not necessarily represent those of their affiliated organizations, or those of the publisher, the editors and the reviewers. Any product that may be evaluated in this article, or claim that may be made by its manufacturer, is not guaranteed or endorsed by the publisher.
